# Dietary Metabolites and Chronic Kidney Disease

**DOI:** 10.3390/nu9040358

**Published:** 2017-04-04

**Authors:** Sho Hasegawa, Tzu-Ming Jao, Reiko Inagi

**Affiliations:** 1 Division of Nephrology and Endocrinology, the University of Tokyo Graduate School of Medicine, Tokyo 113-8655, Japan; shiyohasegawa-tky@umin.ac.jp; 2 Division of CKD Pathophysiology, the University of Tokyo Graduate School of Medicine, Tokyo 113-8655, Japan; d97424006@ntu.edu.tw

**Keywords:** chronic kidney disease, nutrients, uremic toxins, advanced glycated end products, indoxyl sulfate, d-amino acids, palmitate

## Abstract

Dietary contents and their metabolites are closely related to chronic kidney disease (CKD) progression. Advanced glycated end products (AGEs) are a type of uremic toxin produced by glycation. AGE accumulation is not only the result of elevated glucose levels or reduced renal clearance capacity, but it also promotes CKD progression. Indoxyl sulfate, another uremic toxin derived from amino acid metabolism, accumulates as CKD progresses and induces tubulointerstitial fibrosis and glomerular sclerosis. Specific types of amino acids (d-serine) or fatty acids (palmitate) are reported to be closely associated with CKD progression. Promising therapeutic targets associated with nutrition include uremic toxin absorbents and inhibitors of AGEs or the receptor for AGEs (RAGE). Probiotics and prebiotics maintain gut flora balance and also prevent CKD progression by enhancing gut barriers and reducing uremic toxin formation. Nrf2 signaling not only ameliorates oxidative stress but also reduces elevated AGE levels. Bardoxolone methyl, an Nrf2 activator and NF-κB suppressor, has been tested as a therapeutic agent, but the phase 3 clinical trial was terminated owing to the high rate of cardiovascular events. However, a phase 2 trial has been initiated in Japan, and the preliminary analysis reveals promising results without an increase in cardiovascular events.

## 1. Introduction

Chronic kidney disease (CKD) is a significant clinical and public health problem because it is associated with an increased risk of cardiovascular events, hospitalization, and death [1]. Dietary contents and their metabolites are known to be closely related to CKD progression. Accumulation of uremic retention solutes has been observed in patients with CKD [2]. These retained solutes are called uremic toxins when they contribute to uremic syndrome. Patients with progressive CKD must maintain a low potassium and low phosphorus diet [3,4]. As a result, the CKD diet tends to be low in plant fiber and symbiotic organisms, which can alter the normal gut microbiome, leading to overgrowth of bacteria that generate uremic toxins [5]. Uremic toxins, mainly derived from dietary metabolites, are not only the result of kidney failure but also promote the progression of CKD via induction of various pathogenic stress signals [6]. In this review, we focus on nutrition and CKD and summarize recent evidence pertaining to how dietary intake and the resulting metabolites directly or indirectly affect CKD progression. We also discuss promising therapeutic targets associated with nutrition for preventing CKD progression.

## 2. Carbohydrate Metabolism and CKD

**Figure 1:**
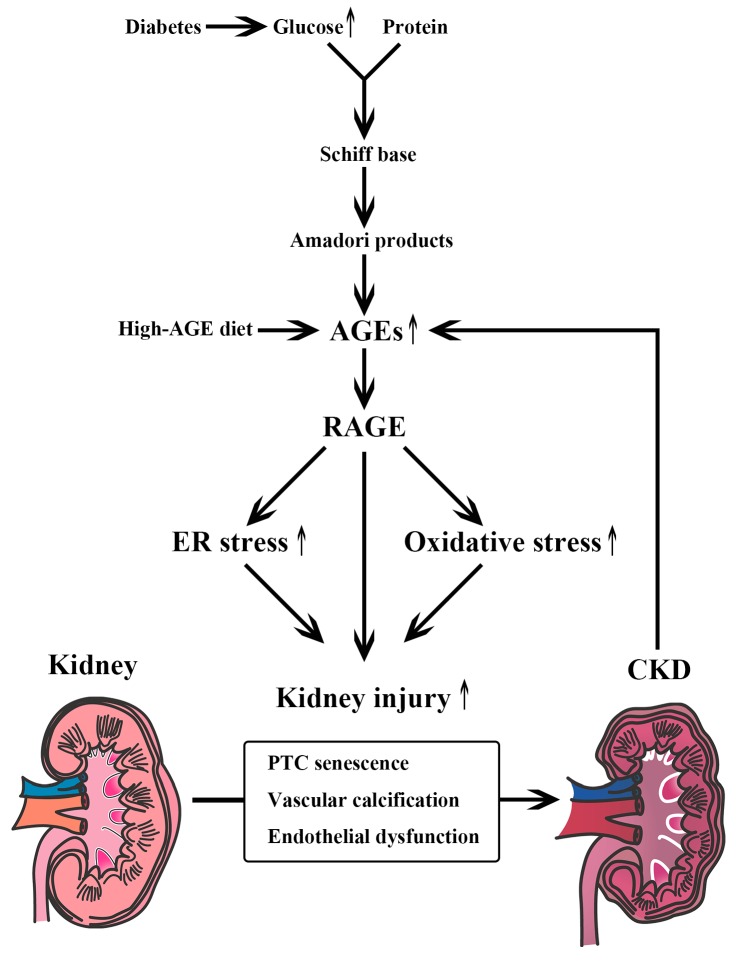
Glycative stress and chronic kidney disease (CKD). Glycative stress caused by uremic toxins, such as AGEs, derived from glycation is closely associated with CKD progression through the activation of the AGE-RAGE system. AGEs; Advanced glycated end products, RAGE; the receptor for AGEs, PTC; proximal tubular epithelial cells.

Chronic hyperglycemia is known to lead various types of proteostasis collapse. Advanced glycated end products (AGEs) are produced by glycation (glycative stress) (Figure 1). Glycation is a non-enzymatic reaction between glucose and proteins that was first described by Maillard in 1912 [7]. First, electrophilic carbonyl groups of glucose react with free amino groups of amino acids, forming a freely reversible Schiff base. Second, Amadori products are formed through rearrangement. Finally, AGEs are produced by oxidation, dehydration, polymerization, and oxidative breakdown of Amadori products [8]. AGEs accumulate in the body when humans are exposed to high levels of glucose, such as in diabetes. AGE levels increase as CKD progresses, as the kidney plays an important role in AGE clearance [9]; renal proximal tubule cells absorb AGEs and catabolize them [10,11]. AGE accumulation is caused not only by decreased clearance but also by endogenous AGE formation or dietary intake. AGE formation can be reduced by cooking with moist heat, using shorter cooking times, cooking at lower temperatures, and using acidic ingredients, such as lemon juice or vinegar [12]. AGEs are stable compounds that are harmful to living organs, including the kidney. In other words, AGEs work as uremic toxins [13]. Vlassara et al. [14] reported that administering AGE-modified rat albumin intravenously resulted in albuminuria and glomerulosclerosis. AGEs are also known to induce vascular calcification and endothelial dysfunction [15,16]. Immunohistochemical studies have shown that AGEs accumulate in the mesangial regions, glomerular capillary walls, and arterial walls of patients with diabetic nephropathy compared to those with healthy kidneys [17,18]. The formation of AGEs is regulated not only by blood glucose levels but also by oxidative stress induced by reactive oxygen species (ROS) [19,20]. As oxidative stress is enhanced in CKD patients, more AGE accumulation occurs [21]. Stimulation of the receptor for AGEs (RAGE) also increases ROS levels through activation of NAPDH oxidase [22] and mitochondrial pathways, which enhances levels of oxidative stress [23,24,25]. Liu et al. [26] reported that the AGE-RAGE system also induces premature senescence of proximal tubular epithelial cells via activation of endoplasmic reticulum (ER) stress-dependent p21 signaling in diabetic nephropathy. 

AGE precursors including methylglyoxal and glyoxal are degraded by enzymes, such as glyoxase-1 (Glo-1), and eliminated from the body through the kidney [15,27]. Some previous reports have shown that activation of Glo-1 produces renal protective effects. Overexpression of Glo-1 in a rat model ameliorated renal ischemia-reperfusion injury by reducing methylglyoxal accumulation and oxidative stress [28]. Overexpressing Glo-1 also suppressed urinary albumin excretion and endothelial dysfunction in a type 1 diabetes model [29]. It has been reported that Glo-1 retards renal senescence by alleviating carbonyl stress and tubular senescence. Ikeda et al. [30] reported that Glo-1 overexpression ameliorated cellular senescence phenotypes in primary renal proximal tubular epithelial cells, while Glo-1 knockdown enhanced these phenotypes. Glo-1-transgenic aged rats also exhibited decreases in senescence markers and in the number of senescence-associated beta-galactosidase positive cells compared to those in wild-type aged rats, which are associated with a reduction in renal AGE accumulation. This suggests that Glo-1 activation can counteract renal tubular senescence induced by glycative stress. Glycative stress is also related to endothelial dysfunction in CKD patients. Endothelium-dependent vasodilation was attenuated and age-related inactivation of endothelial nitric oxide synthase was ameliorated in Glo-1-transgenic rats compared to symptoms in wild-type rats [31]. 

In summary, glycative stress is closely associated with CKD progression through the activation of the AGE-RAGE system or through decreased activity of Glo-1 [32]. 

## 3. Amino Acid Metabolism and CKD

### 3.1. Indoxyl Sulfate, a Metabolite of Tryptophan

**Figure 2:**
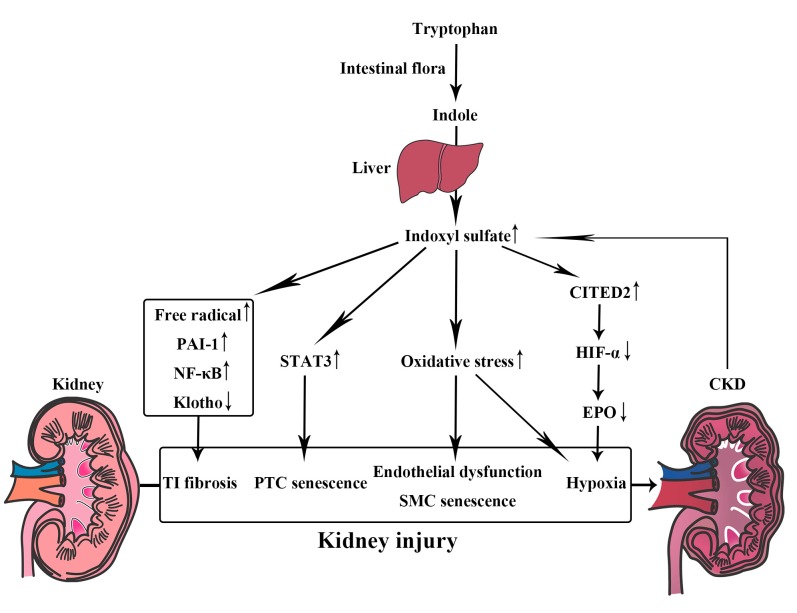
Pathogenic effects of indoxyl sulfate as a uremic toxin. Indoxyl sulfate induces renal tubulointerstitial (TI) fibrosis, renal proximal tubular cell (PTC) senescence, vascular endothelial dysfunction, vascular smooth muscle cell (SMC) senescence, and chronic renal hypoxia, all of which lead to CKD progression. PAI-1; plasminogen activator inhibitor-1, NF-κB; nuclear factor-kappa B, STAT3; signal transducer and activator of transcription 3, CITED2; Cbp/p300-interacting transactivator with Glu/Asp-rich carboxy-terminal domain 2, HIF-α; hypoxia-inducible factor-α, EPO; erythropoietin.

Indoxyl sulfate is a representative uremic toxin derived from amino acid metabolism (Figure 2) [33]. It is metabolized by the liver from indole, which is produced by the intestinal flora as a metabolite of tryptophan [34]. Indoxyl sulfate is excreted from normal kidneys by organic anion transporter 3 [35], but it accumulates as CKD progresses [36]. The accumulation of indoxyl sulfate itself was found to be associated with CKD progression in a prospective observational study [33]. Oral administration of indoxyl sulfate to uremic rats is known to promote the progression of glomerular sclerosis and subsequent renal failure [37]. Indoxyl sulfate also induces various pathogenic phenotypes in human renal proximal tubular HK-2 cells [38,39], such as free radical production, nuclear factor-kappa B (NF-κB) activation, plasminogen activator inhibitor-1 (PAI-1) expression, and suppression of klotho gene expression, all of which lead to tubulointerstitial fibrosis. Indoxyl sulfate increases the enzyme activity of senescence-associated beta-galactosidase, a cellular senescence marker, in HK-2 cells through increased phosphorylation of signal transducer and activator of transcription 3 (STAT3) [40]. In murine proximal renal tubular cells, indoxyl sulfate significantly activates the renal renin-angiotensin-aldosterone system [41]. Accumulation of indoxyl sulfate also induces vascular endothelial cell dysfunction. In human umbilical vein endothelial cells (HUVECs), indoxyl sulfate inhibits endothelial proliferation and reduces endothelial wound repair [42]. Indoxyl sulfate also enhances ROS production, increases NADPH oxidase activity, and significantly decreases levels of glutathione, one of the most active antioxidant systems in HUVECs [43]. 

Indoxyl sulfate is known to stimulate vascular smooth muscle cell proliferation [44] and accelerate vascular smooth muscle cell senescence by increasing oxidative stress [45], which might lead to the progression of arteriosclerosis. Indoxyl sulfate is known to induce chronic tubulointerstitial hypoxia, which acts as a final common pathway leading to end-stage renal disease. Indoxyl sulfate increases oxygen consumption in proximal renal tubules, decreases renal oxygenation, and consequently aggravates hypoxia in the rat kidney, which is dependent on sodium-potassium adenosine triphosphatase and oxidative stress [34]. Indoxyl sulfate also impedes the recruitment of transcriptional coactivators by hypoxia-inducible factor (HIF) via upregulation of Cbp/p300-interacting transactivator with Glu/Asp-rich carboxy-terminal domain 2 (CITED2) through a mechanism of posttranscriptional messenger RNA stabilization [46]; this decreases nuclear accumulation of HIF-α proteins and thereby suppresses HIF target gene expression, such as erythropoietin (EPO) [47]. Via these processes, indoxyl sulfate induces tubulointerstitial fibrosis and glomerular sclerosis, which might lead to CKD progression.

### 3.2. d-Amino Acids

Amino acids exist as one of two enantiomers (d- and l-enantiomers), which are mirror images of each other. In the field of chiral amino acid metabolomics, recent development of techniques to distinguish between d- and l-amino acids has revealed the presence of d-amino acids in living organisms, driving investigations into the cell biology of amino acid chirality.

Pathophysiological activity of d-amino acids has been widely studied in the field of neuroscience before in the field of nephrology. *N*-methyl-d-aspartate receptor (NMDAR)-mediated neurotransmission is vital for learning and memory. Hypofunction of NMDAR has been reported to play a role in the pathophysiology of Alzheimer’s disease. d-amino acid oxidase (DAAO) selectively degrades d-serine, which enhances the activity of NMDAR-mediated neurotransmission [48,49]. A randomized, double-blind, placebo-controlled trial in four major medical centers in Taiwan showed that sodium benzoate, a DAAO inhibitor, substantially improved cognitive and overall functions in patients with early-phase Alzheimer’s disease [50]. 

The pathophysiological activity of d-amino acids has also been found in the field of nephrology. Injection of d-serine has been reported to cause nephrotoxicity in rats; tubular toxicity results from hydrogen peroxide, a metabolite of d-serine degradation by DAAO, which is expressed in the proximal tubular cells [51]. In renal ischemia-reperfusion-injured mice, tubular damage was associated with an increase in serum d-serine [52], while DAAO knockout mice suffered from exacerbation of renal ischemia-reperfusion injuries, indicating that the d-serine metabolites did not cause tubular damage. Thus, there appear to be discrepancies in the mechanism of d-serine nephrotoxicity among species, and the mechanism has not yet been elucidated in humans. There is also a possibility that the d/l ratio of amino acids is associated with CKD progression, as some proteins are known to change natures and structures according to the d/l ratio of amino acids [53]. One study revealed that plasma d-serine levels are elevated in aged individuals or CKD patients [54], and another recently revealed that CKD patients with high d-serine levels carried an approximately 3-fold higher risk of progression to end-stage kidney disease than those with low d-serine levels [55]. Further study is needed to determine the mechanism by which DAAO activity and the d/l ratio of amino acids are associated with CKD progression.

## 4. Fatty Acid Profiles and CKD

It has been reported that plasma phospholipid fatty acid profiles have major effects on living organisms. Palmitate, one of the saturated fatty acids, is known to induce ER stress pathways in vitro [56]. Experiments using rat L6 skeletal muscle cells have shown that mitochondrial DNA damage occurs upstream of palmitate-induced ER stress and autophagy [57].

The role of palmitate in the progression of kidney disease has been demonstrated in several reports. In cultured proximal tubular cells, palmitate increases the expression of monocyte chemoattractant protein-1 (MCP-1) and leads to intracellular accumulation of diacylglycerol (DAG) and subsequent activation of the protein kinase C protein family. This palmitate-induced renal tubular cell damage is attenuated by polyunsaturated fatty acids such as oleate and eicosapentaenoic acids (EPA) [58]. Considering that analogs of orally active epoxyeicosatrienoic acid (EET), the signaling molecule formed by the metabolism of arachidonic acid in the body, attenuate cisplatin-induced nephrotoxicity by reducing oxidative stress, inflammation, ER stress, and apoptosis without affecting the chemotherapeutic effects of cisplatin in experimental rats [59], fatty acid metabolism may be a promising target for preventing CKD progression.

## 5. Metabolic Acidosis and Nutrition

Metabolic acidosis is a common complication of CKD because the kidney plays an important role in maintaining acid-base balance [60]. Metabolic acidosis has various adverse effects, such as increased degradation of muscle protein with muscle wasting, dissolution of bone and bone disease, decreased albumin synthesis, aggravation of inflammation and insulin resistance [60,61]. The data from the Health, Aging, and Body Composition Study, a prospective study of healthy black and white adults of 70–79 years old showed that low serum bicarbonate was associated with higher mortality even for healthy elderly people [62]. When the kidney is exposed to acid, it increases levels of hormones like angiotensin II that help to excrete the acid. The increase in these hormones deteriorates kidney function in the long-term [63]. Therefore, higher dietary acid load can result in metabolic acidosis and is associated with incident CKD and faster kidney disease progression in patients with CKD [64]. Correcting bicarbonate levels by alkali therapy is recommended by NKF-KDOQI (National Kidney Foundation-Kidney Disease Outcomes Quality Initiative) [65] though the target level of serum bicarbonate is still controversial [60,66]. Not only bicarbonate administration but also very low-protein diet, including a diet rich in fruits and vegetables, has been shown to be effective for reducing intake of acids [67].

## 6. Therapeutic Targets Associated with Nutrition for Preventing CKD Progression

### 6.1. Dietary Intake Profile

Dietary AGE is significantly associated with serum AGE accumulation in CKD [68]. AGE intake can be affected not only by ingredients themselves but also by how they are stored and cooked [69]. As mentioned above, the formation of AGEs can be reduced by cooking with moist heat, using shorter cooking times, cooking at lower temperatures, and using acidic ingredients, such as lemon juice or vinegar [12]. A clinical study reported that serum AGEs levels, oxidant stress, and inflammation were reduced in CKD patients after following a low-AGE diet, independently of age [70], suggesting the importance of AGE intake reduction in CKD progression. 

Some early studies suggested that protein restriction might slow progression of CKD [71,72]. Very low-protein diet has been shown to reduce indoxyl sulfate levels [73] and reduce intake of acids [67]. However, according to the Modification of Diet in Renal Disease (MDRD) study, there might be little overall benefit with the low-protein diet [74]. Moreover, a longer-term follow-up of MDRD study showed a higher risk of death in those assigned to the very low-protein group [75]. Overall, the balance of evidence suggests a benefit of moderate dietary protein restriction [76], which is still controversial. 

Some reports have indirectly shown that the quantity and quality of dietary fatty acids affect clinical outcomes in CKD patients, although the pathological findings are various according to the causes of CKD. Reports using dietary assessments suggest that an unfavorable dietary fatty acids pattern (high saturated fatty acid and low polyunsaturated fatty acid intake) is common in patients with CKD [77], which may contribute to clinical outcomes in CKD patients [78]. Although dietary assessment methods are not necessarily accurate [79], fatty acid profiles in the blood are good indicators of habitual dietary fat intake in CKD patients [80]. Based on these evidences, a dietary fatty acids pattern characterized by high polyunsaturated fatty acid and low saturated fatty acid intake may be beneficial for CKD patients.

### 6.2. Uremic Toxin Absorbent (AST-120)

AST-120 (Kremezin; Kureha Chemical, Tokyo, Japan) is an oral, spherical, carbonaceous absorbent. AST-120 can bind many low-molecular-weight compounds (100–10,000 kDa), including indole, the precursor of indoxyl sulfate, in the intestines and prevent indoxyl sulfate production [81]. AST-120 can also bind AGEs such as carboxymethyllysine and decrease serum levels of AGEs in patients with CKD [82]. Moreover, in rat CKD models, AST-120 upregulated renal expression of Nrf2, which plays a protective role in renal injuries, by removing serum indoxyl sulfate [83]. Administration of AST-120 before initiating dialysis has been shown to improve the 1/serum creatinine slope [84] and delay the onset of hemodialysis in CKD patients [85]. However, major multicenter randomized trials conducted in the United States (EPPIC trials) and in Japan (CAP-KD trial) have not supported the benefit of adding AST-120 to standard therapy for CKD patients [86,87]. However, considering that the post-hoc subgroup analysis of the patients from the United States in the EPPIC trials showed that AST-120 delayed the time to dialysis initiation, kidney transplantation, or serum creatinine doubling [88], there is a possibility that AST-120 responders exist among patients with CKD. Further studies are thus needed to identify AST-120 responders.

### 6.3. AGE Formation Inhibitors

The previous experimental data have shown that some compounds trap AGE precursors and attenuate oxidative stress induced by ROS [89]. The B6 vitamer pyridoxamine reduces AGE accumulation by trapping AGE precursors, such as reactive carbonyl and dicarbonyl compounds derived from Amadori compounds [90,91]. This was shown to significantly inhibit increases in albuminuria, plasma creatinine, and hyperlipidemia in diabetic rats without an effect on blood glucose [92,93]. 

Renin-angiotensin system inhibitors, which induce multifunctional renoprotective effects, have been shown to reduce AGE precursors [94,95] and act as antioxidants against AGEs [96]. Hydralazine, another antihypertensive agent that does not interact with the renin-angiotensin system, has also been reported to protect against nephropathy in diabetic rats and decrease AGE formation both in vitro and in vivo [97].

### 6.4. RAGE Inhibitors

Reducing glycative stress via modulation of the AGE-RAGE signal may represent a promising approach for preventing CKD progression [32]. Deletion of RAGE in type 1 diabetic OVE26 mice reduced nephromegaly, mesangial sclerosis, cast formation, glomerular basement membrane thickening, podocyte effacement, and albuminuria [98]. In db/db mice, a neutralizing murine RAGE antibody reduced basement membrane thickness and mesangial volume and attenuated increases in urinary albumin excretion and serum creatinine levels compared with levels in nondiabetic controls [99]. A receptor-based bioabsorbent with an AGE-specific binding capacity has recently been shown to reduce AGEs in serum isolated from end-stage kidney disease patients [100].

### 6.5. Probiotics and Prebiotics

New techniques in metabolomics have shown that alterations in the microbiota profile are closely associated with levels of uremic toxin formation. CKD often alters the microbiota profile in association with an increase in uremic toxins, such as indoxyl sulfate, as disease progresses [101]. Probiotics, bacteria that control the growth of harmful bacteria, maintain gut flora balance and might prevent CKD progression by enhancing gut barriers and reducing uremic toxin formation [102]. Prebiotics, specialized plant fiber that promotes the growth of healthy bacteria in the gut, have also recently been shown to prevent CKD progression. In 5/6 nephrectomy CKD model rats, galacto-oligosaccharides, one type of prebiotics, reduced indoxyl sulfate levels by modifying the microbiota profile; this ameliorated the progression of CKD, which was associated with a reduction in tubular damage caused by ER stress [103]. Lubiprostone also ameliorates the progression of adenine-induced CKD and the accumulation of uremic toxins by improving the gut microbiota and intestinal environment [104]. These data refer to animal research with specific exposure to indoxyl sulfate and cannot be used to generalize the effects, to be sure, but they suggest the possibility that probiotics and prebiotics might prevent CKD progression. 

Mediterranean diet is rich in complex carbohydrates, fibers, vitamins and low in animal proteins and fats, which promotes the beneficial shift to a saccharolytic profile, acting as a selector of good microbes [105,106]. Mediterranean diet has been shown to have the same effects as low protein diet [107] and be associated with lower likelihood of CKD in elderly men [108]. A multiethnic observational study showed that Mediterranean diet was associated with reduced incidence of estimated glomerular filtration rate (eGFR) <60 mL/min/1.73 m^2^ [109]. According to these evidences, mediterranean diet is considered to have the effect of prebiotics.

### 6.6. Nrf2 Pathway Activator

Nrf2 signaling, which regulates genes encoding antioxidant and detoxifying molecules, has been reported to have protective effects in renal failure [110]. Nrf2 signaling not only ameliorates oxidative stress but also induces increased levels of Glo-1 mRNA, protein, and activity, which results in a reduction in AGEs [111]. The activation of Nrf2 signaling would, therefore, be effective in retarding CKD progression. Bardoxolone methyl, an Nrf2 activator, has been tested as a therapeutic agent in clinical trials. The phase 2 clinical trials (BEAM study) showed that bardoxolone methyl increased the eGFR of CKD patients with type 2 diabetes [112]. However, the phase 3 clinical trials (BEACON trial) were terminated because of the high rate of cardiovascular events [113]. However, as the cardiovascular events mainly occurred in patients with increased risk of heart failure at baseline in the BEACON trial and as those of Asian descent generally have fewer cardiovascular events compared with those of Western descent, a phase 2 trial has been initiated in Japan (TSUBAKI study). 

## 7. Conclusions

We have provided an overview of the mechanism by which dietary intake and the resulting metabolites affect CKD progression, as well as discussing promising therapeutic targets associated with nutrition. Most of the promising therapeutic targets, however, have not yet been proved that they are effective in humans. Additional preclinical and clinical studies are needed to identify effective interventions.
